# Supplementary material for: Effectiveness of a Standardized Nursing Process Using NANDA International, Nursing Interventions Classification and Nursing Outcome Classification Terminologies: A Systematic Review
Source: Healthcare (Basel). 2023 Sep 1;11(17):2449. doi: 10.3390/healthcare11172449 (PMC10487812; doi:10.3390/healthcare11172449)
Supplement: Supplementary file 1 [file healthcare-11-02449-s001.zip › File S1.pdf]

1. **Search notebook**

- **Medline (12 January 2021)**

No.	Search strategy	Results
#5	(#4) AND ([english]/lim OR [portuguese]/lim OR [spanish]/lim) AND [1992-2021]/py AND ([medline]/lim OR [pubmed-not-medline]/lim)	719
#4	(#3) AND ([english]/lim OR [portuguese]/lim OR [spanish]/lim) AND [1992-2021]/py	783
#3	#1 OR #2	800
#2	'nursing interventions classification' OR 'nursing outcomes classification' OR 'nanda international' OR 'nnn terminology':ab,ti	800
#1	'nursing interventions classification'/mj OR 'nursing outcomes classification'/mj OR 'nanda international'/mj	67

- **Embase (12 January 2021)**

No.	Search strategy	Results
#5	(#4) AND ([english]/lim OR [portuguese]/lim OR [spanish]/lim) AND [1992-2021]/py AND [embase]/lim	104
#4	(#3) AND ([english]/lim OR [portuguese]/lim OR [spanish]/lim) AND [1992-2021]/py	783
#3	#1 OR #2	800
#2	'nursing interventions classification' OR 'nursing outcomes classification' OR 'nanda international' OR 'nnn terminology':ab,ti	800
#1	'nursing interventions classification'/mj OR 'nursing outcomes classification'/mj OR 'nanda international'/mj	67

- **SCI-EXPANDED (12 January 2021)**

No.	Search strategy	Results
#2	TEMA: ('nursing interventions classification' OR 'nursing outcomes classification' OR 'nanda international' OR 'nnn terminology') OR TÍTULO: ('nursing interventions classification' OR 'nursing outcomes classification' OR 'nanda international' OR 'nnn terminology')<br>Refinado por: IDIOMAS: (ENGLISH OR PORTUGUESE OR SPANISH) AND ÁREAS DE INVESTIGACIÓN: (NURSING)<br>Índices=SCI-EXPANDED Período de tiempo=Todos los años	593
#1	TEMA: ('nursing interventions classification' OR 'nursing outcomes classification' OR 'nanda international' OR 'nnn terminology') OR TÍTULO: ('nursing interventions classification' OR 'nursing outcomes classification' OR 'nanda international' OR 'nnn terminology')<br>Índices=SCI-EXPANDED Período de tiempo=Todos los años	1.509

- **SCIELO (12 January 2021)**

No.	Search strategy	Results
#2	TEMA: ('nursing interventions classification' OR 'nursing outcomes classification' OR 'nanda international' OR 'nnn terminology') OR TÍTULO: ('nursing interventions classification' OR 'nursing outcomes classification' OR 'nanda international' OR 'nnn terminology')<br>Refinado por: ÁREAS DE INVESTIGACIÓN: (NURSING)<br>Índices=SCIELO Período de tiempo=Todos los años	272
#1	TEMA: ('nursing interventions classification' OR 'nursing outcomes classification' OR 'nanda international' OR 'nnn terminology') OR TÍTULO: ('nursing interventions classification' OR 'nursing outcomes classification' OR 'nanda international' OR 'nnn terminology')<br>Índices=SCIELO Período de tiempo=Todos los años	294

- **Cochrane (12 January 2021)**

No.	Search strategy	Results
#1	("nursing interventions classification" OR "nursing outcomes classification" OR "nanda international" OR "nnn terminology"):ti,ab,kw (Word variations have been searched)"	31
#2	MeSH descriptor: [Standardized Nursing Terminology] this term only	1
#3	#1 OR #2<br>with Cochrane Library publication date from Jan 1992 to Jan 2021	31

- **CINAHL (12 January 2021)**

No.	Search strategy	Results
S3	S1 OR S2 **Limitadores** - Fecha de publicación: 19920101-20210131; Idioma: English, Portuguese, Spanish	(1,326)
S2	TI (‘nursing interventions classification' OR 'nursing outcomes classification' OR 'nanda international' OR 'nnn terminology') OR AB ('nursing interventions classification' OR 'nursing outcomes classification' OR 'nanda international' OR 'nnn terminology')	(1,291)
S1	(MM "NANDA International")	(158)

- **Scopus (12 January 2021)**

No.	Search strategy	Results
#1	TITLE-ABS-KEY ("nursing interventions classification” OR “nursing outcomes classification” OR “nanda international” OR "nnn terminology") AND (EXCLUDE (PUBYEAR , 1985)) AND (LIMIT-TO (SUBJAREA , "NURS")) AND (LIMIT-TO (LANGUAGE , "English") OR LIMIT-TO (LANGUAGE , "Portuguese") OR LIMIT-TO (LANGUAGE , "Spanish"))	688

- **LILACS 13 (January 2021)**

No.	Search strategy	Results
#1	(mh:("Standardized Nursing Terminology" OR "Nursing Diagnosis/classification" OR "NANDA International")) OR (ti:(NANDA OR "Diagnostico enfermero" OR "Standardized Nursing Terminology")) OR (ab:(NANDA OR "Diagnostico enfermero" OR "Standardized Nursing Terminology"))	722

- **Tripdatabase (12 January 2021)**

56 result for "nursing interventions classification” OR “nursing outcomes classification” OR “nanda international” OR "nnn terminology
