# Supplementary material for: Effectiveness of a Standardized Nursing Process Using NANDA International, Nursing Interventions Classification and Nursing Outcome Classification Terminologies: A Systematic Review
Source: Healthcare (Basel). 2023 Sep 1;11(17):2449. doi: 10.3390/healthcare11172449 (PMC10487812; doi:10.3390/healthcare11172449)
Supplement: Supplementary file 1 [file healthcare-11-02449-s001.zip › File S2 (test notebook).pdf]

Testing notebook

EMTREE (Embase):

Nursing terminology: EMTREE term (Embase); MeSH term (Medline): “Standardized Nursing Terminology”

Note: I would use these which are more specific and have to do more specifically with the study:

- **NANDA international:** NANDA taxonomy; NANDA Classification; NANDA Diagnoses; NANDA Diagnose; NANDA Nursing Diagnoses; NANDA Nursing Diagnose; NANDA Terminologie; NANDA-I (NANDA international)
- **Nursing outcomes classification:** NOC (nursing outcomes classification); nursing outcome classification
- **Nursing interventions classification:** NIC (nursing interventions classification); nursing intervention classification

Possible terms drawn from the results of different tests (authors' keywords, database suggestions, etc.):

- outcome assessmen,
- health care management,
- health care planning,
- health care policy,
- home health care classification,
- information processing,
- interdisciplinary communication,
- international classification for nursing practice,
- medical education,
- nursing care,
- nursing diagnosis,
- nursing interventions classification,
- nursing outcomes classification,
- nursing research,
- Omaha system,
- patient care,
- perioperative nursing

Embase Session Results (1 Feb 2020)

TEST 1:

#4	#2 AND #3	**1248**
#3	nurs*:ab,ti	**524287**
#2	nanda OR nic OR noc:ab,ti	**29521**
#1	'nanda international':ab,ti OR nic:ab,ti OR noc:ab,ti	**4398**

TEST 2:

#3	#1 OR #2	**458**
#2	'nursing interventions classification' OR 'nursing outcomes classification' OR 'nanda international':ab,ti	**450**
#1	'nursing interventions classification'/mj OR 'nursing outcomes classification'/mj OR 'nanda international'/mj	**45**

TEST: 3

Note: I did the same as in test 2 but added the terms "NNN terminology" and "NNN". It retrieved much more than double the previous results, I did a new test including only these two terms (row #4. I am sending a spreadsheet with the first 100 references, in case you want to check what comes out).
As there is so much difference between the results that include these 2 terms (row #3) and those that do not (row #3 of TEST 2), I decided to check what would happen if I removed from the results all together, the references related to these 2 terms (row #4). There are about 200 references that come up even if the terms in row #4 are not used, suggesting that they are relevant references; the rest up to 909 could be noise (non-relevant references). As there were so many, I decided to do another check: I selected, from the results of row #4, a reference addressing the NANDA-NIC-NOC topic row #6 and asked for all references in row #3 except this row #7. Positive result. This suggests to me that we should remove the acronym "NNN", because reviewing the results of #4, I found that this acronym is also used for other terms such as "National neuroblastoma network" and "N′-nitrosonornicotine".

#7	#3 NOT #6	**1619**
#6	'cross-mapping of nursing care terms recorded in italian hospitals into the standardized nnn terminology':ti	**1**
#5	#3 NOT #4	**711**
#4	'nnn terminology' OR nnn:ab,ti	**909**
#3	#1 OR #2	**1620**
#2	'nursing interventions classification' OR 'nursing outcomes classification' OR 'nanda international' OR 'nnn terminology' OR nnn:ab,ti	**1351**
#1	'nursing interventions classification'/mj OR 'nursing outcomes classification'/mj OR 'nanda international'/mj	**45**

<u>Embase Session Results (3 Feb 2020)</u>

TEST: 4

Note: I took the outcomes group terms from the PICO structure and used them to narrow down the results of row #3 (I removed "NNN" from the search equation). It seems to me that they restrict too much... We could give a further twist to the terms in the group of rows #3-#6 or, considering that there is not too much on NANDA-NIC-NOC, use only these terms (row #3).

#8	#3 AND #7	**20**
#7	OR/ #4-6	**66969**
#6	'nursing intervention'/mj	**520**
#5	((diagnostic OR intervention) NEAR/1 accuracy):ab,ti	**61336**
#4	'diagnostic accuracy'/mj	**11732**
#3	#1 OR #2	**727**
#2	'nursing interventions classification' OR 'nursing outcomes classification' OR 'nanda international' OR 'nnn terminology':ab,ti	**727**
#1	'nursing interventions classification'/mj OR 'nursing outcomes classification'/mj OR 'nanda international'/mj	**45**
