# Supplementary material for: Effectiveness of a Standardized Nursing Process Using NANDA International, Nursing Interventions Classification and Nursing Outcome Classification Terminologies: A Systematic Review
Source: Healthcare (Basel). 2023 Sep 1;11(17):2449. doi: 10.3390/healthcare11172449 (PMC10487812; doi:10.3390/healthcare11172449)
Supplement: Supplementary file 1 [file healthcare-11-02449-s001.zip › Tables S1-S3.pdf]

Table S1: Interobserver agreement on included studies

Author (year)	Kappa coefficient	p-value	Confidence Interval
Corcoles et al. (2021) [12]	1.000	0.003	1.000 – 1.000
Guerra et al. (2021) [13]	0.270	0.230	-0.191 –0.731
Lemos et al. (2020) [25]	0.571	0.058	0.098 – 1.045
Rembold et al. (2020) [26]	0.357	0.284	-0.361 – 1.076
Silva et al. (2020) [16]	0.357	0.284	-0.361 – 1.076
Bjorklund-Lima et al. (2019) [24]	0.609	0.047	-0.057 – 1.274
Pascoal et al. (2019) [17]	1.000	0.003	1.000 – 1.000
Silva et al. (2019) [18]	1.000	0.003	1.000 – 1.000
Vázquez-Sánchez et al. (2019) [27]	0.088	0.708	-0.297 – 0.047
Gencbas et al. (2018) [14]	1.000	0.003	1.000 – 1.000
Sampaio et al. (2018) [15]	1.000	0.003	1.000 – 1.000
Pascoal et al. (2016) [19]	0.088	0.708	-0.297 – 0.047
Reis & Jesus (2015) [20]	0.088	0.708	-0.297 – 0.047
Pascoal et al. (2014) [21]	0.727	0.023	0.242 – 1.212
Laguna-Parras et al. (2013) [28]	1.000	0.003	1.000 – 1.000
Cárdenas-Valladolid et al. (2012) [22]	0.127	0.571	-0.423 – 0.076
Müller-Staub et al. (2008) [23]	1.000	0.003	1.000 – 1.000

Table S2: Critical reading scores for the included studies

Author (year)	Methods	Q1	Q2	Q3	Q4	Q5	Q6	Q7	Q8	Q9	Q10	Q11	Score
Corcoles et al. (2021) [12]	RCT	Y	Y	Y	Y	Y	Y	NA	NA	Y	Y	Y	9/11
Guerra et al. (2021) [13]	RCT	Y	Y	Y	Y	Y	Y	NA	NA	Y	Y	Y	9/11
Lemos et al. (2020) [25]	Quasi-experimental	Y	N	Y	N	N	Y	NA	NA	Y	Y	Y	6/11
Rembold et al. (2020) [26]	Case control	Y	Y	Y	Y	Y	D	NA	NA	Y	Y	D	7/11
Silva et al. (2020) [16]	Cohort	Y	Y	Y	Y	Y	NA	NA	Y	Y	Y	N	8/11
Bjorklund-Lima et al. (2019) [24]	Cohort	Y	Y	Y	Y	Y	NA	NA	Y	N	Y	Y	8/11
Pascoal et al. (2019) [17]	Cohort	Y	Y	Y	D	Y	NA	NA	Y	Y	Y	N	7/11
Silva et al. (2019) [18]	Quasi-experimental	Y	N	Y	N	N	Y	NA	NA	Y	Y	Y	6/11
Vázquez-Sánchez et al. (2019) [27]	RCT	Y	Y	D	Y	Y	Y	NA	NA	Y	Y	Y	8/11
Gencbas et al. (2018) [14]	Pseudo RCT	Y	Y	Y	Y	Y	Y	NA	NA	Y	Y	Y	9/11
Sampaio et al.	RCT	Y	Y	Y	Y	Y	Y	NA	NA	Y	Y	Y	9/11

(2018) [15]												
Pascoal et al. (2016) [19]	Cohort	Y	Y	Y	D	Y	NA	NA	Y	Y	Y	N	7/11
Reis & Jesus (2015) [20]	Cohort	Y	Y	Y	D	Y	NA	NA	Y	D	Y	N	6/11
Pascoal et al. (2014) [21]	Cohort	Y	Y	Y	D	D	NA	NA	Y	N	Y	Y	6/11
Laguna-Parras et al. (2013) [28]	Quasi-experimental	Y	N	Y	N	N	Y	NA	NA	Y	Y	Y	6/11
Cárdenas-Valladolid et al. (2012) [22]	Cohort	Y	Y	D	Y	Y	NA	NA	Y	Y	Y	Y	8/11
Müller-Staub et al. (2008) [23]	RCT	Y	Y	Y	D	Y	Y	NA	NA	Y	Y	Y	8/11
Percentage Y		100	8.33	88.8	55.5	77.7	90	NA	100	88.8	100	70,5	

RCT/Quasi-experimental (Q1: Was the trial guided by a clearly defined question?; Q2: Was the allocation of patients to treatments random?; Q3: Were all the patients who took part adequately considered until completion of the study?; Q4: Was blinding maintained?; Q5: Were groups similar on study commencement?; Q6: Were the groups treated equally regardless of the study intervention?; Q7: Was the treatment effect large?; Q8: How accurate was this effect?; Q9: Can these results be applied to your setting or local population?; Q10: Were all clinically important results taken into account?; Q11: Do the benefits obtained justify the risks and costs?)
Cohort (Q1: Was the study focused on a clearly defined topic?; Q2: Was the cohort recruited in the most appropriate way?; Q3: Was the outcome measured accurately with the aim of minimizing potential bias?; Q4: Did the authors take into account the potential effect of confounding factors in the design and/or analysis of the study?; Q5: Was subject follow-up complete and of sufficient duration?; Q6: What are the results of the study?; Q7: How accurate are the results?; Q8: Do the outcomes appear credible?; Q9: Are the study results in line with other available evidence?; Q10: Can the results be applied in your setting?; Q11: Is this going to alter your clinical decision?)
Case-control (Q1: Was the study focused on a clearly defined topic?; Q2: Did the authors use an appropriate method to respond to the question?; Q3: Were the cases recruited/included in an acceptable way?; Q4: Were the controls selected in an acceptable way?; Q5: Was the exposure accurately measured to minimize possible biases?; Q6: What confounding factors did the authors take into account?; Q7: Did the authors take the potential for confounding factors into account in the design and/or analysis?; Q8: What is the accuracy of the results? What is the accuracy of the risk estimate?; Q9: Do you find the results credible?; Q10: Can the results be applied in your setting?; Q11: Are the results of this study in line with other available evidence?)
Y: Yes; N: No; NA: Not applicable.

Table S3. JBI level of evidence and degree of certainty using GRADE methodology

Author; year	Methods	Initial certainty	Risk of bias	Inconsistency	Indirectness	Imprecision	Publication bias	Magnitude of effect	Response gradient	Residual confounding	JBI evidence level	Final certainty
Corcoles et al. (2021) [12]	RCT	++++	0	0	0	0	0	1	1	0	1c	++++
Guerra et al. (2021) [13]	RCT	++++	-1	0	0	-1	0	0	0	0	1c	++
Lemos et al. (2020) [25]	Quasi-experimental	++++	0	0	0	-1	0	0	0	0	2c	+++
Rembold et al. (2020) [26]	Case control	++	-1	0	0	0	0	1	1	0	3d	+++
Silva et al. (2020) [16]	Cohort	++	0	0	0	0	0	1	1	0	3e	++++
Bjorklund-Lima et al. (2019) [24]	Cohort	++	0	0	0	0	0	0	0	0	3e	++
Pascoal et al. (2019) [17]	Cohort	++	0	0	0	0	0	1	1	0	3e	++++
Silva et al. (2019) [18]	Quasi-experimental	++++	-2	0	0	0	0	1	1	0	2c	++++
Vázquez-Sánchez et al. (2019) [27]	RCT	++++	0	0	-1	-1	0	0	0	0	1c	++
Gencbas et al. (2018) [14]	Pseudo RCT	++++	-1	0	-1	-1	0	0	0	0	1d	+
Sampaio et al. (2018) [15]	RCT	++++	-1	0	-1	-1	0	0	1	0	1c	++
Pascoal et al. (2016) [19]	Cohort	++	0	0	0	0	0	1	1	0	3e	++++
Reis & Jesus (2015) [20]	Cohort	++	0	0	0	0	0	1	1	0	3e	++++
Pascoal et al. (2014) [21]	Cohort	++	0	0	0	0	0	1	1	0	3e	++++
Laguna-Parras et al. (2013) [28]	Quasi-experimental	++++	-1	0	0	0	0	0	0	0	2d	+++
Cárdenas-Valladolid et al. (2012) [22]	Cohort	++	0	0	-1	0	0	1	0	0	3c	++
Müller-Staub et al. (2008) [23]	RCT	++++	0	0	-1	-1	0	0	0	0	1c	++
